# Association between markers of brain pathology and verbal memory binding in autosomal dominant Alzheimer’s disease

**DOI:** 10.1002/alz.092260

**Published:** 2025-01-09

**Authors:** Mario A Parra‐Rodriguez, Ana Y Baena, Diana Munera, Alex L Badillo‐Cabrera, Nikole A Bonillas Félix, Francisco Lopera, Yakeel T. Quiroz

**Affiliations:** ^1^ University of Strathclyde, Glasgow UK; ^2^ Grupo de Neurociencias de Antioquia, Universidad de Antioquia, Medellín Colombia; ^3^ Harvard Medical School, Boston, MA USA; ^4^ Massachusetts General Hospital, Harvard Medical School, Boston, MA USA; ^5^ Grupo de Neurociencias de Antioquia, Universidad de Antioquia, Medellin Colombia; ^6^ Harvard Medical School, Massachusetts General Hospital, Boston, MA USA; ^7^ Neurosciences Group of Antioquia, University of Antioquia, Medellín Colombia

## Abstract

**Background:**

There is a need to develop neuropsychological measures to detect Alzheimer’s disease (AD)‐pathology and track cognitive changes along the AD trajectory. We previously showed that memory decline during word list learning (CERAD), associative memory (LAS‐FNAME test), working memory (Visual Short‐Memory Binding Test), and others, are associated with amyloid and tau pathology in members of Colombian families with autosomal dominant AD. Here we sought to determine whether associative verbal memory (Memory Binding Test ‐ MBT, (Buschke, 2014)) is associated with PET in vivo markers of brain pathology and whether it can distinguish those who will develop dementia later in life due to autosomal‐dominant AD from age‐matched non‐carriers.

**Methods:**

A total of 33 PSEN1‐E280A carriers (Age=37.6±6.17; 61% females) and 37 non‐carriers (Age=35.1±5.87; 54% females) from the Colombia‐Boston (COLBOS) Biomarker study were included. They were matched according to age and education. All participants completed cognitive testing, florbetapir (amyloid), and flortaucipir (tau) PET imaging. We implemented regression models to investigate the extent to which group membership, cortical amyloid burden, and regional tau pathology accounted for performance on the MBT.

**Results:**

Carriers showed poorer performance on the MMSE (p<0.001) and higher scores on the daily living functions scale (FAST, p<0.001), suggesting they were in the early symptomatic stages. Performance on the MBT was significantly accounted for by group membership (R^2^=15.7%, p < 0.001) whereby carriers of the mutation exhibited significant deficits (p<0.001). Aβ and Tau pathology also accounted for performance on the MBT with the best model retaining cortical Aβ and Tau in the precuneus (R^2^=54.7%, p < 0.001). Controlling for disease severity (MMSE and FAST), but not for age or education, removed the above effects.

**Conclusions:**

Associative memory (i.e., MBT) is sensitive to AD‐related brain pathology in the early stages of ADAD due to PSEN1‐E280A. These current findings, together with those reported previously, suggest that the early stages of the ADAD continuum can be traced with sensitive memory tests.

